# Effects of four days of isokinetic training on isometric and isokinetic peak torque and muscle cross-sectional area in women

**DOI:** 10.1080/15502783.2025.2550162

**Published:** 2025-08-29

**Authors:** Jocelyn E. Arnett, Dolores G. Ortega, Trevor D. Roberts, Justin S. Pioske, Robert W. Smith, Tyler J. Neltner, Richard J. Schmidt, Terry J. Housh

**Affiliations:** a University of Nebraska – Lincoln, Lincoln, NE, USA; b Lindenwood University, St. Charles, MO, USA; c University of Wisconsin – Platteville, Platteville, WI, USA

**Keywords:** Strength, isokinetic, hypertrophy, women

## Abstract

**Background:**

Short-term resistance training provides insights into the early phase adaptations of muscular strength and muscle cross-sectional area (CSA). The purpose of this study was to examine the effects of 2, 3, and 4 days of maximal, concentric, reciprocal isokinetic forearm extension (FE) and forearm flexion (FF) training at 180°∙s^−1^ on peak torque (PT) during maximal voluntary isometric contractions (MVIC) and isokinetic muscle actions at 60, 180, and 300°∙s^−1^, and changes in muscle CSA in untrained women.

**Methods:**

Twelve women (Mean ± SD: age: 21.7 ± 1.2 yrs; height: 167.9 ± 5.2 cm; body mass: 64.7 ± 6.8 kg) completed 4 days of maximal, concentric, reciprocal isokinetic FE and FF training at 180°∙s^−1^ for 6 sets of 10 repetitions with the non-dominant arm (based on throwing preference). In addition, the subjects completed 4 testing visits (Pre-testing and following 2 [Day2], 3 [Day3], and 4 [Day4] days of training) with the non-dominant arm that included 2 FE and 2 FF MVICs (elbow at 90°), followed by 3, maximal, concentric, reciprocal isokinetic FE and FF muscle actions at 60°∙s^−1^, 180°∙s^−1^, and 300°∙s^−1^. Changes in PT were assessed using separate 4 (Velocity: MVIC, 60°∙s^−1^, 180°∙s^−1^, and 300°∙s^−1^) ×4 (Time: Pretest, Day2, Day3, and Day4) repeated measures ANOVAs for FE and FF. Changes in CSA were assessed using separate 2 (Muscle: Triceps Brachii and Biceps Brachii) ×4 (Time: Pretest, Day2, Day3, and Day4) repeated measures ANOVAs. An alpha value of *p* ≤ 0.05 was used for all ANOVAs. Follow-up pairwise comparisons were used when necessary.

**Results:**

For FE PT (decomposed by Velocity), Day2 (28.0 ± 8.3 Nm;*p* = 0.036, *d* = 0.563) and Day4 (28.4 ± 9.3 Nm;*p* = 0.042, *d* = 0.572) MVIC were greater than Pre-test (23.9 ± 6.1 Nm), and at 60°∙s^−1^, Day4 FE PT was greater (24.3 ± 9.2 Nm;*p* = 0.015, *d* = 0.304) than Day2 (21.8 ± 7.1 Nm). For FF PT (collapsed across Velocity), Day3 (29.6 ± 6.2 Nm;*p* = 0.006, *d* = 0.0.190) and Day4 (31.1 ± 6.8 Nm;*p* < 0.001, *d* = 0.409) were greater than Day2 (28.4 ± 6.4 Nm), and Day3 (*p* = 0.018, *d* = 0.377) and Day4 (*p* < 0.001, *d* = 0.593) were greater than Pretest (27.3 ± 6.0 Nm). In addition, Day4 FF PT (31.1 ± 6.8 Nm) was greater (*p* = 0.007, *d* = 0.231) than Day3 (29.6 ± 6.2 Nm). For CSA (collapsed across Muscle), Day2 (4.56 ± 0.75 cm^2^;*p* = 0.006, *d* = 0.408), Day3 (4.59 ± 0.73 cm^2^;*p* = 0.004, *d* = 0.455), andDay4 (4.61 ± 1.0 cm^2^;*p* = 0.025, *d* = 0.400) were greater than Pretest (4.27 ± 0.69 cm^2^).

**Conclusions:**

A minimum of 3 days of isokinetic training is needed for untrained women to see improvements in isometric and isokinetic strength and increases in the CSA of the non-dominant arm.

